# Global Hand: Time to Capacity Build

**DOI:** 10.29252/wjps.9.3.349

**Published:** 2020-09

**Authors:** Murtaza Kadhum, Pierre Sinclair, Roba Khundkar

**Affiliations:** 1 NDORMs, Botnar Research Centre, University of Oxford, UK;; 2 Roba Khundkar, NDS, John Radcliffe Hospital, University of Oxford, UK

**Keywords:** Global surgery, Hand surgery, Plastic surgery, Orthopedics, Training, Education

**DEAR EDITOR**

In 2015, the Lancet Commission defined and estimated the global burden of surgical disease; revealing 33% of the overall global burden of disease as surgical in origin and estimating that 95% of the population in low and middle income countries (LMICs) lack basic surgical care.^1^ As a means to rectify this, the commission agreed upon various targets, including improving access to timely surgery, workforce density and overall surgical volume.^2^ With this came growing interest in improving global provision of hand surgery. In 2013, the previous editor of the Journal of Hand Surgery (European Volume) called for further action globally, especially in improving research capacity in LMICs.^3^ *But, where are we now? *

Today, various organizations exist that target hand conditions ranging from congenital malformations to injury. Notably, these include ReSurge International and the Touching Hands Program (THP), who aim to provide direct surgical care and local training of surgeons. At the time of writing this letter, the latter claim to have conducted 41 missions in 13 countries.^4^ However, these programmes remain limited by their ability to provide suitable patient-reported outcomes, surgical results and long-term efficacy of their training provision. 

Global outreach in hand surgery may follow differing models; the vertical approach or direct supply remains the most common internationally, but fails to build long-term sustainability. A horizontal approach that builds infrastructure is less common, usually due to the need for significant government-level support. The gold standard approach theorizes a combination of vertical and horizontal approaches, aiming to build local infrastructure, inter-professional training and enhance research capacity. This synergy remains difficult to implement, due to deficiencies in financial support, government willpower and differing priorities of relevant stakeholders. 

The overarching difficulty in global hand surgery provision remains that lack of appropriate and local surgical training programmes. Without formal training programmes developing interest in hand surgery as a subspecialty, LMICs will continue to struggle to provide long-term sustainable surgical provision. Developing local training programmes will enable timely access to essential surgery, increase specialist hand surgeon density and surgical volume, whilst minimizing perioperative mortality – thereby contributing to the targets set out by the Lancet Commission. Examples of such efforts can be seen through existing models such as the College of Surgeons of East, Central and Southern Africa (COSECSA) and 2^nd^ Chance partnership. 

This Swiss organization provides planned and curricula driven support for COSECSA training programmes in reconstructive surgery including hand surgery. The focus is solely on training of local surgeons, especially those on COSECSA training programmes. 

Although surgical missions and local training of healthcare professionals are commendable, the authors suggest a shift in outreach priorities, focusing on developing locally run and nationally and internationally supported hand surgery training programmes within LMICs, involving all relevant stakeholders including the local governing bodies, academic institutes and professional bodies. 

## CONFLICT OF INTEREST

The authors declare no conflict of interest.
